# Intention to use eight antenatal care model and associated factors among pregnant women who come for antenatal care in Amhara Region Referral Hospitals, Ethiopia

**DOI:** 10.1016/j.heliyon.2025.e42633

**Published:** 2025-02-11

**Authors:** Eyuel Amare, Azmeraw Ambachew Kebede, Endeshaw Admassu, Samuel Kefelegn, Anteneh Gashaw

**Affiliations:** a Clinical Midwifery Professional at Amhara Regional Health Bureau, Amhara region, Ethiopia; b School of Midwifery, College of Medicine & Health Sciences, University of Gondar, Gondar, Ethiopia; c Department of Midwifery, College of Medicine & Health Sciences, Dilla University, Dilla, Ethiopia

**Keywords:** Ethiopia, Intention, Eight antenatal care, Contact, Amhara

## Abstract

**Background:**

Maternal and perinatal mortality is highly associated with four or fewer antenatal care (ANC) visit. Due to this, WHO develop a new model called minimum of eight antenatal care (ANC8+) contact. This model is used to re-think and re-design the service through context specific expectations. Besides, the model is beyond survival, aim to maximize health and wellbeing of women even at post pregnancy motherhood, and social health of the family and community at large. Since shortage of related studies in Ethiopia so far, this study aim to address those gaps.

**Objective:**

To assess intention to use ANC8+ model and associated factors among pregnant women who come for antenatal care contact in Northwest of Amhara region referral hospitals, Ethiopia, 2021.

**Method:**

An institution-based cross-sectional study was conducted from September 1st to October 30th, 2021 in Northwest of Amhara regional state referral hospitals. A multistage sampling technique was used to select 847 eligible women. A structured, pretested, and interviewer-administered questionnaire was employed. EPI data version 4.6 and SPSS version 23 were used for data entry and analysis, respectively. Binary logistic regression model was fitted to identify factors associated with pregnant women intention to use ANC8+ model. A p-value of ≤0.05 was used to declare statistical association in the last model.

**Result:**

Overall, 739 (88.8 %, 95 % CI: 86.68, 90.97) women had the intention to use ANC8+. Pregnant women in the age group between 20 and 30 years (AOR = 19.84, 95 % CI: 3.01, 130.64), and ≥31 years (AOR = 3.92, 95 % CI: 1.79, 8.58), being farmer in occupation (AOR = 0.13, 95 % CI: 0.04, 0.43), government employee (AOR = 5.58, 95 % CI: 1.64, 18.97), good attitude (AOR = 38.76, CI 5.99, 250.73), positive subjective norm (AOR = 10.83, 95 % CI: 4.36, 26.92) and perceived behavioral control (AOR = 8.98, 95 % CI: 3.26, 24.71) were factors associated with women's intention to use ANC8+.

**Conclusion:**

More than four-fifths of the pregnant women had intention to use ANC8+. Increase in age, being the government employee, having good attitude, positive subjective norm and perceived behavioral control were positively, while being a farmer negatively associated to intention to use ANC8+ contact. Therefore, empowering women, developing socio cultural integrity with the health system through health education and trainings to local health cadres and community leaders would possibly increase the intention of using ANC8+.

## 1 Introduction

Antenatal care (ANC) is defined as routine care provided by skilled health professionals to pregnant women between conception and initiation of labor to ensure good pregnancy conditions [1]. It is also the gateway to integrated care, promote healthy practice, and ensure referral linkage to high risk pregnancies [2].

Globally, more than 295,000 women die annually due to pregnancy related complications in 2017 [3]. Besides, the magnitude of perinatal mortality is continued to be higher across the world [4]. The maternal mortality ratio is still higher in Sub Saharan Africa (SSA) [5]. Likewise, Ethiopia has a huge burden of perinatal [4] and maternal mortalities as a devastating challenge as well. The maternal mortality ratio (MMR) in Ethiopia is estimated to be 412 in 2016 [6].

Since maternal and perinatal morbidity and mortality are still increasing, the new global agenda that is called to be Sustainable Development Goal (SDG) is endorsed to be effective till 2030. Specifically, SDG target 3 aims to reduce the global maternal mortality ratio to less than 70 per 100,000 live births and as low as the neonatal mortality to 12 per 1000 live births [7]. Hence, ANC is better to decrease those problems by filling the gap in the continuum of care by early diagnosis, treatment, and prevent pregnancy related difficulties [1]. According to empirical evidence the perinatal mortality is found to be high in those who lack antenatal care [1,2,8,9].

The utilization of ANC is low across the globe. Hence, around 71 % of pregnant women receive ANC across the world [10]. Besides, about 69 % and 44 % of pregnant women attended first and fourth ANC visits respectively in SSA [10]. While, 62 % pregnant women received ANC for once in Ethiopia [6]. Previously, the World Health Organization (WHO) recommended all risk free pregnant women to have at least four goal oriented clinic centered ANC visits, called focused ANC [1]. Since 2016, however, WHO recommends all pregnant women to have a minimum of eight ANC contacts. The minimum of eight antenatal care (ANC8+) is the antenatal care given for pregnant women at least eight times per single pregnancy time [1]. Besides, the word ‘visits’ has been changed by ‘contact’ which is to increase close interaction between health professionals and pregnant women rather than simply follow up number increment [1]. In this regard, the first ANC contact should be takes place before 12 weeks of gestation, two contacts in the second trimester (at 20 and 26 weeks), and five contacts in the third trimester (at 30,34,36,38 and 40 weeks of gestation) [11]. This newly emerged ANC8+ contacts recommendation is designed to maximize the quality of ANC and make better outcome for maternal, fetal and newborns. Because previously the fetal death in between 32 and 36 weeks of gestation was higher with goal oriented and reduced antenatal care package [12]. Not only had this but also mattered for the post pregnancy motherhood and social health. Despite the previous four visit focused ANC model, the new WHO ANC model promotes a more active connection between ANC clients to their health care providers. Midwife-led continuity of care, community based intervention, early ultrasound scanning, medical problem screening such as tuberculosis, gestational diabetes mellitus and other cultural and socio demographic context specific related issues will be address by this new model under the concept of positive pregnancy experience [13].

Intention has the most immediate influence on behavior to perform antenatal care contact through maintaining women's self-regulation. Because of the women's self-regulation increment, the use of antenatal care service will be assured [14]. So, having intention has positive and unidirectional effect on antenatal care utilization. If intention increased so that the antenatal care utilization increased. Pregnant women with strong intention to use ANC8+ tends to actual practicing of ANC8+ at the ground level [15]. In turn, intention is directly affected by the individual's attitude, subjective norm, and perceived behavioral control [16]. Hence, pregnant women's intention to the newly endorsed ANC8+ is an important concern for effective practice of the new antenatal care model. Even though the ANC8+ recommendation was launched in 2016, Ethiopia accepted the model recently [15] and developed the ANC guideline under the WHO 2016 recommendation [15]. In a study carried out in northwest Ethiopia regarding the utilization of ANC8+ contacts, it was found that only 20.9 % of individuals took advantage of this program [17]. Hence, women's intention is the mainstay to effectively implement the model thereby achieving the desired goals. Therefore, this study aimed to assess pregnant women's intention to use the ANC8+ model and associated factors in referral hospitals of northwest Amhara regional state.

## 2 Methods

### 2.1 Study design, setting and period

An institution-based cross sectional study was conducted from September 1st to October 30th, 2021. The study was conducted in the Northwest of Amhara regional state referral hospitals. The Amhara region is located at the Northwest Ethiopia. Northwestern part the region consists of six zones that contains a total population of 13,049,742. From those females account about 6,501,156 [18]. In the Northwest part of the Amhara region, there are 5 referral hospitals such as: the University of Gondar comprehensive and specialized Hospital, Felegehiwot comprehensive and specialized hospital, Tibebe Gion comprehensive and specialized Hospital, Debretabor comprehensive and specialized Hospital and Debremarkos comprehensive and specialized Hospital. Each referral hospital's catchment population is estimated to be 5–7 million people [15]. According to recent report, the average monthly flow of women for first antenatal care service is around 200–250 for each referral hospital as evidenced from hospitals monthly report [19].

### 2.2 Source and study population

#### 2.2.1 Source population

All pregnant women who came for antenatal care contact in Northwest of Amhara regional state referral hospitals.

#### 2.2.2 Study population

All pregnant women who came for antenatal care contact in selected referral hospitals of Northwest Amhara regional state during the study period.

#### 2.2.3 Inclusion criteria

All pregnant women who came for antenatal care contact before 16 week of gestation in selected hospitals of Northwest Amhara regional state had included.

#### 2.2.4 Sample size determination

The sample size was determined by using the single population proportion formula by considering the following assumptions: proportion of intention to use ANC8+ 50 % (since there was no previous study), 95 % confidence level, and 5 % margin of error.

$$n=\frac{{\left(Z\alpha /2\right)}^{2}*P\left(1‐p\right)}{d^{2}}$$

Therefore,

$$n=\frac{{\left(1.96\right)}^{2}*0.5\left(1-0.5\right)}{\left(0.05\right)2}=385$$

Where; n = the desirable sample size, Z = standard normal distribution curve value for 95 % confidence level = 1.96, α = level of significance, P = proportion of intention to use ANC8+, and d = 5 % margin of error. By considering a 10 % none response rate and a design effect of 2 (since two stage sampling), the minimum adequate sample size was 847.

### 2.3 Sampling procedure and technique

There are five referral hospitals in Northwest of Amhara regional state. A multi-stage sampling technique was employed to select hospitals for a total of 847 eligible women. In the first stage, 60 % [3] hospitals were selected randomly by lottery method including, the Felegehiwot comprehensive specialized hospital, Debretabor comprehensive specialized hospital, and Debremarkos comprehensive specialized hospital. The monthly first ANC contact flow was obtained from the respective hospitals and the skipping interval was computed. Thus, the total average client flow from the three selected hospitals was 2022 by three month divided by the calculated sample size (847) resulted in 2.3 (approximated to 2). The study participants then selected every 2nd interval. 320 participants were chosen from Felegehiwot Comprehensive Specialized Hospital, 250 observations were selected from Debretabor Comprehensive Specialized Hospital, and 277 observations were selected from Debremarkos Comprehensive Specialized Hospital. The first case was selected randomly. Thereafter, the calculated sample size was proportionally allocated to each hospital. Then, systematic random sampling technique was used to select the study participants from the respective hospitals.

### 2.4 Study variables

#### 2.4.1 Outcome variable

Intention to use eight antenatal care model (intended or not intended).

### 2.5 Independent variables

Socio demographic factors: Age, residence, average household monthly income, occupation, educational status, media exposure and marital status. Husband/male partner related factors: Husband/male partner involvement, husband occupation, husband educational status and household decision-making power Reproductive history and maternal health Service related factors: Distance to health institution, gravidity, parity, knowledge of pregnancy danger sign, previous experience to ANC, previous pregnancy outcome, pregnancy planeness and perceived interaction Behavioral and social factors: Attitude, subjective norm, and perceived behavioral control.

### 2.6 Operational definition

#### 2.6.1 Intention to use ANC8+ model

An indication of pregnant women readiness/willingness to use ANC8+ and how much an effort they were planning to exert, and to use ANC8+. It was measured by using four questions. Each question contains of five points Likert scale. Finally, the outcome variable was dichotomized and women who scored ≥60 % (point score ≥12) considered as intended while those who scored <60 % (Point score <12) considered as not intended to use ANC8+ [10].

### 2.7 Exposure to media

Respondents were asked if they listen radio, watch television, or read magazine. Those who respond at least once a week were considered to be regularly exposed to that form of media [6].

### 2.8 Husband/male partner involvement

Measured using five variables with a yes/no response. A score of 1 was given for ‘’Yes'’ and 0 for ‘’No’’. The involvement score for each respondent was ranged from 0 = no involvement to 5 = involved in all 5 activities. From total, women who scored of 3–5 was considered as a '’High'’ male involvement while those who scored 0–2 was considered as '’Low'’ relative to this particular population [20].

### 2.9 Perceived interaction

The health care provider to client relationship was measured by 13 questions on a 5-point Likert scale; the total score was ranged from 13 to 65. To group participants by their level of the perceived health care provider to client relationship, participants were divided into three groups; a score of 13–51 was considered to be ‘low’, a score of 52–64 ‘medium’, and a score of 65 was considered to be an ‘optimal’ perceived relationship [21].

### 2.10 Knowledge of pregnancy danger signs

In this research, a woman was considered as knowledgeable, those mentioned at least three key danger signs of pregnancy [22].

### 2.11 Women's attitude toward ANC8+

Attitude towards ANC8+ was measured using four questions. Each question contained five point Likert scales (1 = strongly disagree, 2 = disagree, 3 = neutral, 4 = agree, 5 = strongly agree). The total score was between 4 and 20 and women who scored ≥60 % (point score ≥12) considered as having good attitudes [21].

### 2.12 Subjective norms (SN)

Subjective norm towards ANC8+ utilization, related to women perceptions of social pressure to utilize ANC8+ measured by using five questions. Each question had contained five points Likert scales (1 = strongly disagree, 2 = disagree, 3 = neutral, 4 = agree, 5 = strongly agree). The total score was 5–25 and women whose scored ≥60 % (point score ≥15) considered as having favorable SNs [21].

### 2.13 Perceived behavioral control (PBC)

Perceived behavioral control towards ANC8+ is an indicator of the extent to which women feel confident to utilize ANC8+. PBC was measured by using three questions. Each question had contained five point Likert scales (1 = strongly disagree, 2 = disagree, 3 = neutral, 4 = agree, 5 = strongly agree). The total score was ranged from 3 to 15 and women who scored ≥60 % (point score ≥9) considered as having positive PBC [21].

### 2.14 Data collection tools and procedures

The data collection tool was developed by reviewing literature and guidelines (7,54–57) and data were collected using a structured, interviewer administered questionnaire through face-to-face interviews. The content validity of the questionnaire was judged by a group of researchers who are experts on maternal and child health to evaluate and enhance the items in the question. Therefore, some corrections were done accordingly. The questionnaire contains socio-demographic, husband/male partner related factors, reproductive history and maternity health service, and social and behavioral related characteristics. Three BSc and three MSc Midwives, were trained at common place, about the interview technique, and how to collect and supervise the data respectively. Data were collected from the women directly after she took the antenatal care service at the same day of interview, immediately after she leaved the service room. The data collectors used the ANC registration to identify interview candidate pregnant women. Gestational age estimation was done either from the last normal menstrual period, early ultrasound, or pregnancy fundal height.

### 2.15 Data quality control

Initially, the questionnaire was prepared in English and translated to Amharic language and back to English by two different language experts with the help of health professionals to ensure its consistency and better understandability. Two days training was given on how to collect the data, the way of approach to the study participants, interview technique, and how to keep information. A week before the actual data collection, a pretest was done on pregnant women of Tibebe Gion specialized hospital on 5 % (43) of the sample size to check response, language clarity, and appropriateness of the questionnaire. Thereafter, amendments done accordingly. Order rearrangements done and ambiguity words replaced. During the actual data collection, the questionnaire was checked for completeness daily by the supervisors.

### 2.16 Data processing and analysis

The collected data were checked for completeness manually and incomplete data were excluded from the analysis. Then, the data were checked, coded, and entered into EPI data version 4.6 statistical software and then exported to SPSS version 23 for analysis. Descriptive statistics like percentages, frequency, mean, standard deviation stated using tables and graphs to present the characteristics of study participants. Chi-squared test was performed to examine the association between individual-level factors and the outcome variable. The binary logistic regression model was fitted to identify risk factors for pregnant women intention to use the ANC8+ recommendation. Initially, bivariable analysis was done to identify the candidate explanatory variables for the multivariable analysis. Thereafter, all explanatory variables having a p-value of ≤0.25 in the bivariable analysis was included in the multivariable logistic regression analysis to handle the effect of possible confounders and to identify independent factors affecting pregnant women intention to use the ANC8+. The model fitness also cheeked using Hosmer – Lemishow goodness-fit-test and it yields a p value of 0.077 and backward variable selection method were used, **the level of significance was declared based on a 95 % CI at a p-value of <0.05.**

## 3 Result

### 3.1 Socio demographic characteristics of the respondents

Eight hundred and thirty two women were participated in this study with a response rate of 98.2 %. The mean age of the respondent was 27.47 years (SD ± 4.88). Three hundred sixty six (44 %) were between the age group of 25–29 years. More than three-quarters of the respondents (76.6 %) were Orthodox Christians. Three hundred and twenty three respondents (38.8 %) had completed college and above education. The greater part (95.1 %) of the study participants were married. Most of the respondents (95.7 %) were urban dwellers. More than two-thirds (69.8 %) of the study participants had an average monthly income of ≥5000 ETB-(Table 1).

**Table 1:** Socio demographic characteristics of pregnant women who came for antenatal care in Northwest of Amhara region referral hospitals, Ethiopia, 2021 (n = 832).

Variable	Category	Frequency	Percent
**Age in years**	≤20	60	7.2
	21–30	594	71.4
	≥31	178	21.4
**Religion**	Orthodox	637	76.6
	Muslim	153	18.4
	Protestant	38	4.5
	Catholic	4	0.5
**Educational status**	unable to read and write	99	11.9
	able to read and write	30	3.6
	Primary education [[1], [2], [3], [4], [5], [6], [7], [8]]	119	14.3
	Secondary education [[9], [10], [11], [12]]	261	31.4
	College and above	323	38.8
**Marital status**	Married	798	95.9
	Not married	34	4.1
**Residence**	Urban	796	95.7
	Rural	36	4.3
**Income (ETB)**	≤1499	69	8.3
	1500–3499	78	9.4
	3500–4999	104	12.5
	≥5000	581	69.8
**Occupational status**	House wife	317	38.1
	Farmer	29	3.5
	Merchant	148	17.8
	Student	26	3.1
	Government employee	213	25.6
	Self-employee	89	10.7
	Job-finder	10	1.2
**Husband educational status (n=793)**	Unable to read and write	31	3.9
	Able to read and write	56	7.0
	Primary education [[1], [2], [3], [4], [5], [6], [7], [8]]	87	11.0
	Secondary education [[9], [10], [11], [12]]	158	19.9
	College and above	461	58.2
**Husband occupational status (n=793)**	Farmer	44	6.0
	Merchant	137	17.0
	Student	4	0.5
	Government employee	406	51.0
	Self-employee	202	25.5
**Mass media exposure**	Yes	761	91.5
	No	71	8.5
**How far from the health institution**	>60 min	259	31.1
	≤60 min	573	68.9
**Means of transport**	On foot	267	32.1
	By taxi	536	64.4
	Public transport	29	3.5

### 3.2 Behavioral and social factors

**Fig. 1:**
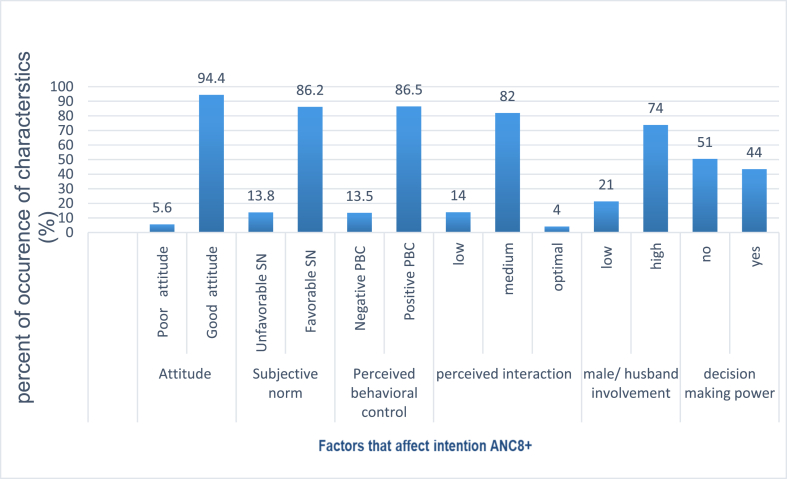
Factors that affect intention to use ANC8+ among pregnant women who came for antenatal care in Northwest of Amhara region referral hospitals, Ethiopia, 2021.

In this section house hold decision making power, husband/male partner involvement, perceived interaction between health professional and pregnant women and other behavioral factors had described. From the total respondents almost half (50.5 %) had no house hold decision making power. Most respondents (95.1 %) had husband/male partner involvement. Regarding to the perceived interaction between health care provider and pregnant women more than four-fifths (82 %) of the respondents had medium interaction. Among the respondents, seven hundred and eighty five respondents (94.4 %) had good attitude. While, 86.2 % participants had favorable to subjective norms (Fig. 1).

### 3.3 Maternal health service and reproductive health related factors

There were four hundred and ninety six respondents that had prior pregnancy, from these less than 5 % were knowledgeable to pregnancy danger sign. The 59.5 % respondents were multi gravida, among those multigravida women, 90.5 % had previous ANC experience, and the rest no history of follow up. Their main reason (70 %) was no information associated with ANC follow up (Table 2).

**Table 2:** Obstetrics and reproductive health related factors that affect intention to use ANC8+ among women who came for antenatal care in Northwest of Amhara region referral hospitals, Ethiopia, 2021.

Variable	Category	Frequency	Percent
**Gravidity (n=832)**	Primi gravida	337	40.5
	Multi gravida	495	59.5
**Previous ANC experience (n=495)**	Yes	448	90.5
	No	47	9.5
**Prior ANC visit (n=448)**	<4 times	99	22.1
	≥4 times	349	77.9
**Reason not to attend the ANC (n=47)**^a^	No information	33	70.2
	Has no use	11	23.4
	Others	13	27.6
**Pregnancy plan ness (n=832)**	Planned	755	90.7
	Not planned	77	9.3
**Number of previous pregnancy (n=495)**	One	160	32.3
	Two	172	34.8
	Three	105	21.2
	Four	44	8.9
	≥ Five	14	2.8
**Parity (n=495)**	Null para	23	4.7
	Multi para	472	95.3
**Pregnancy outcome (n=495)**^a^	Abortion	88	17.7
	IUFD	48	9.6
	Alive	456	90.12
	Neonatal loss	2	0.4
**Knowledge of pregnancy danger signs (n=832)**	Knowledgeable	46	5.5
	Not knowledgeable	786	94.4

a does not sum up to the total due to possibility of multiple response.

### 3.4 Pregnant women's intention to use ANC8+ and associated factors

**Fig. 2:**
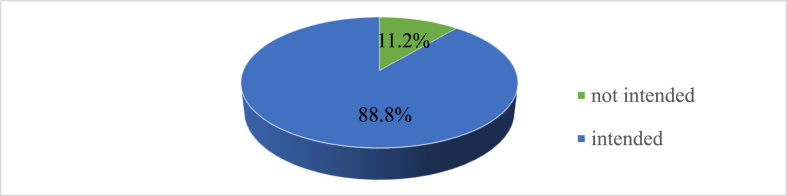
Prevalence of intention to use ANC8+ among pregnant women who came for antenatal care contact in Northwest of Amhara region referral hospitals, Ethiopia, 2021.

In this study, about 88.8 % (95 % CI: 86.68, 90.97) were intended to use eight antenatal care model (Fig. 2). The bi-variable and multivariable logistic regression analysis were fitted to identify associated factors with the outcome variable. Thus, in the bivariable logistic regression, age, residence, education, occupation, house-hold monthly income, marital status, attitude, subjective norm, perceived behavioral control, perceived interaction between the health professional and the pregnant women, mass media exposure, and the distance from home to the health institution were factors associated with intention to use ANC8+.

Women with the age group of between 20 and 30 and ≥31 years old had higher odds of intention to use ANC8+ as compared to those <20 years (AOR = 19.84, 95 % CI: 3.01, 130.64), (AOR = 3.92, 95 % CI: 1.79, 8.58) respectively. Farmer pregnant women (AOR = 0.13, 95 % CI: 0.04, 0.43) were 87 % less likely intended to use the service as compared to house-wives. While, those under government employee were 5.58 times more likely intended to use ANC8+ (AOR = 5.58, 95 % CI: 1.64, 18.97). Accordingly, the study established that those women with positive subjective norm and perceived behavioral control were 10.83 (AOR = 10.83, 95 % CI: 4.36, 26.92) and 8.98 (AOR = 8.98, 95 % CI: 3.26, 24.71) times more likely intended to use ANC8+ respectively as compared to their counter-parts (Table 3).

**Table 3:** Bi-variable and multivariable logistic regression analysis of factors associated with pregnant women's intention to use ANC8+ model, Northwest of Amhara region referral hospitals, Ethiopia, 2021 (n = 832).

Variables	Category	Intention to use ANC8+		COR (95 %CI)	AOR (95 %CI)
		Yes	No		
Age in years	≤20	58	2	1	1
	21–30	546	48	0.39 (0.09, 1.65)	19.84 (3.01, 130.64) ∗
	≥31	135	43	0.10 (0.02, 0.46)	3.92 (1.79, 8.58) ∗∗
Residence	Urban	717	79	5.77 (2.84, 11.73)	2.75 (0.38, 19.89)
	Rural	22	14	1	1
Education	Unable to read and write	63	36	1	1
	Able to read and write	28	2	8.00 (1.80, 35.56)	1.55 (0.19, 12.21)
	primary school [[1], [2], [3], [4], [5], [6], [7], [8]]	110	9	6.98 (3.15, 15.44)	0.66 (0.15, 2.92)
	secondary school [[9], [10], [11], [12]]	241	20	6.88 (3.73, 12.71)	0.91 (0.23, 3.51)
	college and above	297	26	6.52 (3.68, 11.57)	0.91 (0.17, 4.70)
Occupation	house wife	273	44	1	1
	Farmer	19	10	0.30 (0.13, 0.70)	0.13 (0.04, 0.43) ∗∗
	Merchant	140	8	2.82 (1.29, 6.15)	2.03 (0.63, 6.48.)
	Student	23	3	1.23 (0.35, 4.28)	0.51 (0.10, 2.51)
	government employee	198	15	2.12 (1.15, 3.93)	5.58 (1.64, 18.97) ∗
	Self-employee	77	12	1.03 (0.52, 2.05)	0.77 (0.25, 2.36)
	job finder	9	1	1.45 (0.17, 11.73)	2.07 (0.11, 38.20)
House hold monthly Income (ETB)	≤1499	49	20	1	1
	1500–3499	70	8	3.57 (1.45–8.76)	1.22 (0.20, 7.41)
	3500–4999	87	17	2.08 (1.00–4.35)	0.20 (0.03, 1.06)
	≥5000	533	48	4.53 (2.49–8.24	0.43 (0.10, 1.76)
Marital status	Married	73	85	2.58 (1.13, 5.88)	1.88 (0.31, 11.21)
	Not married	26	8	1	1
Attitude	Good	737	48	345.46 (81.35, 1467.10)	38.76 (5.99, 250.73) ∗∗
	Poor	2	45	1	1
Subjective norm	Favorable	693	25	40.04 (23.21, 69.09)	10.83 (4.36, 26.92) ∗∗
	Unfavorable	47	68	1	1
Perceived behavioral control	Positive	687	33	24.02 (14.42, 39.99)	8.98 (3.26, 24.71) ∗∗
	Negative	52	60	1	1
Perceived interaction between professional and women	Low	108	8	1	1
	Medium	604	78	0.57 (0.26, 1.22)	1.02 (0.17, 5.80)
	Optimal	27	7	0.28 (0.09, 0.85)	0.25 (0.05, 1.11)
Mass media	Exposed	691	70	4.73 (2.71, 8.23)	1.15 (0.31, 4.17)
	Not exposed	48	23	1	1
Time taken from home to health institution	<60 min	527	46	2.54 (1.64, 3.93)	1.20 (0.51, 2.82)
	≥60 min	212	47	1	1
Pregnancy plan ness	Planned	678	77	2.31 (1.26, 4.20)	1.37 (0.49, 3.76)
	Unplanned	61	16	1	1
Gravidity	Primi gravid	313	24	2.11 (1.29, 3.43)	1.32 (0.51, 3.44)
	Multi gravid	426	69	1	1

Note: ∗significant at p value < 0.05, ∗∗ significant at p value ≤ 0.001, AOR = adjusted odd ratio, COR = crud odd ratio, 1 reference.

## 4 Discussion

This study assessed the intention of pregnant women to use the ANC8+ model and associate factors among women who came for antenatal care contact in Northwest of Amhara region referral hospitals. After five years (since 2016) of WHO new ANC8+ model recommendation, Ethiopia adopted recently and contextualized to the local setting so as to support achieving goal of ending preventable maternal and perinatal death by 2030 [15]. By considering this outcome of interest the current study was the foremost in-country level to assess intention of pregnant women to ANC8+.

The result of this study revealed that about 88.8 % of the pregnant women have had intention to use ANC8+ contact. This finding is lower than a study done in Kenya 99.4 % [23]. The difference in the health system policy and, the hierarchical passage in the implementation of health related agenda across those countries could be attributed to make the difference. Nevertheless, the current study finding is higher than the study done in Sehala Seyemit district in Amhara region, Ethiopia (62.7 %) [24]. The difference might came from the study area related issues, in which the current study only less than 5 % respondents were from the rural area, while Sehala Seyemit is remote and too far to reach to health institutions and even poor schooling, So that their educational status and information access would be challenging. Hence, these constraints might limit the women intention to use ANC8+. Besides, getting better ANC service comparable to urban residents may be limited. This related with previous antenatal care service quality, good service by itself might also have positive effect on women intention building for next ANC correlated service. The better achievement of the minimum of 8 antenatal care service in urban area than rural was supported by studies done in Nigeria, Benin and Ethiopia [2,[25], [26], [27]]. Besides, since the current study done at health institution, the intention of women who already came to health institution to seek care would definitely good than those women at home who were assessed with community based cross sectional design. Perhaps, the financial constraint could also take its share and going to make a difference. Because urban dwellers are financially secured than those in rural setting [28].

Women's age was found to be significant factor for intention to use ANC8+. In this study, older women (between 20 and 30, and greater than 31 years old) were 19.84 and 3.92 times more likely intended to use ANC8+ as compared to their younger counters. This study was consistent with local study in northern and north west of Ethiopia [29,30]. The possible justification might be as the age increase, so does the better knowledge, understanding, and experience of the pre-pregnancy and pregnancy time preparation. With the age increase, appreciating the need of antenatal care service could addressed very well through life experience. This might positively influence the women to develop a certain intention on antenatal care service.

This study revealed that being government employee was found to be significant predictor of intention to use ANC8+ contact. Hence, government employed women were 5.58 times more likely intended to use ANC8+ contact compared to those who were house-wives. Previous study done in Nigeria shows that most (96.5 %) antenatal care service users were employed women [31]. This supports the current study finding, that the women who had intention to use ANC8+ were employed either with government or private sector. Government employee were socioeconomically better to manage wisely. This is important to maintain cost to transportation, medication and other service related wastes to antenatal care. Television and other social media instruments are important to gain information and develop good intention to antenatal care service [31]. The study also showed that farmer pregnant women were 87 % less likely intended to use the service as compared to house-wives. A study from Ghana show pregnant women intention to a new eight antenatal care model was too low among poorest as compared to richest [32]. Besides, from the country context most of the rural dwellers are farmers, comparably not considered as economically good to manage appropriately. So such like reasons might affect the women's intention to use ANC8+.

Women react to the antenatal care service environment through envisioning the future plan. This women intention overtly leads to poor or good attitude, which is the basic frame to antenatal care service. Since attitude is a psychological tendency, expressed by evaluating certain event with some degree of favor or disfavor. In this study those with having positive attitude had higher odds of intention to use minimum of eight ANC. This was supported by the study done in Indonesia [16]. Furthermore, under this study those who have positive attitude were 38.76 times more likely intended to use ANC8+. The finding was greater than the study done in rural setting [21,24]. Women in urban area can improve health care literacy. Hence, those women might prone to good attitude, and more of appreciative to intention. Women in rural area may be influenced by sociocultural norms that had effect on the antenatal care service related attitude building.

This study showed that subjective norm was found to be significant predictor of intention to use ANC8+ contact. Those women who had favorable subjective norm were 7.2 times more likely intended to use ANC8+ as compared to those women who had unfavorable subjective norm. The finding is consistent with other studies [24]. Furthermore, the current finding is concomitantly stable with the 2015 WHO antenatal care guideline steering committee suggestion, that recognized the need of maximizing the number of antenatal care contacts and put the need of women from ANC with their view, cultural beliefs and expectations. For instance, the main reason to shift from focused antenatal care visit to eight antenatal care contact was the women want and need of positive pregnancy experience across the world continents. This positive pregnancy experience mainly rest on the social norm, cultural and socio demographic contexts [14].

In addition, evidence revealed that the family, friend or social support strongly increase intention. The support could have many directions either informational assist and advice or emotional support like attention and affection to the service of ANC. Moreover, the instrumental support either with material or time related support were valuable to influence intention positively [16]. Some pregnant women needs motivation to seek antenatal care, otherwise, they might simply focus on their work. Pregnancy by itself has stress effect, especially on Primi-gravida women. Perhaps, the social support might relief related concerns.

This study revealed that perceived behavioral control was found to be significant predictor of intention to use ANC8+ contact. Those women who had positive perceived behavioral control were nearly 6 times more likely intended to use ANC8+ as compared to those who had negative perceived behavioral control. This was in line with one subtheme of the positive pregnancy experience stating achieving positive motherhood, its principle is developing maternal self-esteem, competence, and autonomy [14].

### 4.1 Strength and limitation

The major strength of this study is that it has become the foremost at the population-based study on ANC8+ in Ethiopia. Therefore, it can serve as stimulus and benchmark for the next studies. Despite the contribution of the study to the literature on maternal health care, this study has some limitations. First, it is a cross-sectional study in which temporal relations could not be assessed. There could be social desirability bias that lead to exaggerated prevalence of intention since the women asked immediately after the service given. Beliefs of the women toward intentions to use ANC8+ may not addressed since lack of theory of planned behavioral approach.

## 5 Conclusion

More than four-fifths of the pregnant women had intention to use ANC8+. Factors such as occupation, age, Attitude, subjective norm, and perceived behavioral control were significantly associated to the intention to use ANC8+ contact. Therefore, empowering women, developing socio cultural integrity through health education and trainings to local health cadres and respected body would possibly increase the intention of using ANC8+. Since health cadres are elected by the community and considered as human resource of the community, responsible and respected throughout carrying the process of health related operations, and means to cooperate to health workers, family leaders, to tackle problem and inform the new government health agendas.

## CRediT authorship contribution statement

**Eyuel Amare:** Writing – original draft, Software, Methodology, Investigation, Funding acquisition, Formal analysis, Data curation, Conceptualization. **Azmeraw Ambachew Kebede:** Visualization, Validation, Supervision, Software, Project administration, Methodology. **Endeshaw Admassu:** Visualization, Validation, Supervision, Project administration, Methodology. **Samuel Kefelegn:** Writing – original draft, Methodology, Formal analysis, Conceptualization. **Anteneh Gashaw:** Writing – review & editing, Writing – original draft, Visualization, Validation, Supervision, Software, Resources, Project administration, Methodology, Investigation, Funding acquisition, Formal analysis, Data curation, Conceptualization.

## Ethical approval and consent to participate

The ethical approval was obtained from the University of Gondar's School of Midwifery's ethical review committee with reference number (SMIDW/23/2014), A letter of permission was written to the Amhara regional health bureau from the school of midwifery, University of Gondar. The letter of cooperation was written by Amhara regional health bureau for each hospitals. Following an explanation of the purpose of the study, written informed consent was obtained from each participants. For those who cannot write and read the informed consent form was read in front of witness (their friends, relatives and independent body of research team) and the witness also sign the informed consent form, so for those minor participants the informed written consent was taken from their parents. Also, affirmation was made that they are free to withdraw consent and discontinue participation without any form of prejudice. All participants were assured confidentiality of information and privacy of their personal information. To preserve confidentiality, the data was not exposed to any third party except the investigators. All necessary methods were carried out in accordance with the guidelines of institutional and declaration of Helsinki.

## Consent for publication

Not applicable.

## Data availability statement

The datasets generated and/or analyzed during the current study are not publicly available(the data that has been used is confidential) due to preserving participant anonymity but are available from the corresponding author on reasonable request (Anteneh Gashaw, antenehgashaw77@gmal.com).

## Funding

Amhara regional health beauro provided funds for the data collection and stationary materials of this research work with a project grant code of Acct. No GOV-1000264750214. The website of the university is www.uog.edu.et. "The funders had no role in study design, data collection, and analysis, decision to publish, or preparation of the manuscript."

## Declaration of competing interest

The authors declare the following financial interests/personal relationships which may be considered as potential competing interests: Anteneh Gashaw reports financial support was provided by Amhara National Regional Health Bureau.

## List of Abbreviations

ANC: Antenatal Care
ANC8+: Minimum of 8 Antenatal Care
PBC: Perceived Behavioral Control
SDG: Sustainable Development Goal
